# Multi-proxy constraints on Atlantic circulation dynamics since the last ice age

**DOI:** 10.1038/s41561-023-01140-3

**Published:** 2023-04-03

**Authors:** Frerk Pöppelmeier, Aurich Jeltsch-Thömmes, Jörg Lippold, Fortunat Joos, Thomas F. Stocker

**Affiliations:** 1 Climate and Environmental Physics, Physics Institute, University of Bern, Bern, Switzerland; 2 Oeschger Centre for Climate Change Research, University of Bern, Bern, Switzerland; 3 Institute of Earth Sciences, Heidelberg University, Heidelberg, Germany

**Keywords:** Palaeoceanography, Marine chemistry, Climate and Earth system modelling, Palaeoclimate

## Abstract

Uncertainties persist in the understanding of the Atlantic meridional overturning circulation and its response to external perturbations such as freshwater or radiative forcing. Abrupt reduction of the Atlantic circulation is considered a climate tipping point that may have been crossed when Earth’s climate was propelled out of the last ice age. However, the evolution of the circulation since the Last Glacial Maximum (22–18 thousand years ago) remains insufficiently constrained due to model and proxy limitations. Here we leverage information from both a compilation of proxy records that track various aspects of the circulation and climate model simulations to constrain the Atlantic circulation over the past 20,000 years. We find a coherent picture of a shallow and weak Atlantic overturning circulation during the Last Glacial Maximum that reconciles apparently conflicting proxy evidence. Model–data comparison of the last deglaciation—starting from this new, multiple constrained glacial state—indicates a muted response during Heinrich Stadial 1 and that water mass geometry did not fully adjust to the strong reduction in overturning circulation during the comparably short Younger Dryas period. This demonstrates that the relationship between freshwater forcing and Atlantic overturning strength is strongly dependent on the climatic and oceanic background state.

## Abstract (web summary)

The Atlantic meridional overturning circulation was shallow and weak during the Last Glacial Maximum, and water masses took time to adjust to circulation shifts during the Last Deglaciation, according to a reassessment of proxy records and model simulations.

## Main

Recent studies suggest that the Atlantic meridional overturning circulation (AMOC), a critical constituent of inter-hemispheric redistribution of heat and carbon, transitioned to an anomalous weak state since the mid-twentieth century^1,2^ and could approach a tipping point towards collapse even within the twenty-first century^3,4^. Reconstructions of the AMOC suggest that this tipping point may have previously been crossed multiple times since the Last Glacial Maximum (LGM; 20 thousand years ago (ka))^5^, with consequences recorded globally^6^. The two strongest of these events occurred from 17.5 to 14.7 ka and from 12.9 to 11.7 ka (Heinrich Stadial 1 (HS1) and Younger Dryas (YD), respectively) and were associated with pronounced northern hemispheric cold spells^7^ while atmospheric temperatures increased in the Southern Hemisphere^8^. This bipolar seesaw is thought to be linked to the abrupt decline of the northerly ocean heat transport concomitant with a strong AMOC^9,10^. Consequently, the bipolar seesaw is responsible for the potential disruptive global-scale impacts during major changes in Atlantic overturning.

However, no clear picture has yet emerged on the exact changes of the AMOC during these past events, and proxy-based reconstructions suggest vastly different manifestations, from no major weakening^11,12^ to full collapse^5,13^ of the circulation. A major challenge with reconstructions of past AMOC is that interpretations are often based only on a subset of the available proxies, which are all tracing different aspects of the AMOC such as water mass provenance or advection strength^14^ while they are additionally spatio-temporally limited. This issue is further exacerbated by the inherent ambiguities of the proxies as they are subject to the influence of multiple environmental factors that are particularly hard to disentangle in a data-driven approach alone. Model simulations, by contrast, invoke various mechanisms to generate millennial-scale variability in agreement with certain climate and ocean proxies^15–17^, yet a fully coherent framework is still lacking. Therefore, the evidence for past AMOC threshold behaviour remains uncertain and limits our understanding of this critical constituent of Earth’s climate system.

In this Article, we employ an Earth system model of intermediate complexity equipped with four major isotope-based water mass tracers to first establish an LGM circulation state that reconciles apparently conflicting proxy evidence through direct model–data comparison. We then use this as a starting point for a suite of transient simulations of the last 20 kyr that exhibit a range of different AMOC evolutions. This permits us to evaluate the impact of rapid halts and resumptions of the overturning circulation, or the lack thereof, on the simulated palette of proxies. Through comparisons of the simulated with reconstructed proxy time series in this dynamically and biogeochemically consistent framework, we are able to comprehensibly assess the intensity of the disruptions in Atlantic overturning during the YD and HS1 and evaluate their impact on key ocean characteristics.

## Constraints on last glacial AMOC

We use the Bern3D model to evaluate 51 LGM ocean states that differ in water mass geometry and overturning strength and test their ability to reproduce the Atlantic distributions of proxy reconstructions. The dynamically simulated and therefore internally consistent four major ocean-circulation proxies are benthic stable carbon isotopes (δ^13^C)^18^, benthic minus planktic radiocarbon age (B-P age)^18^, neodymium isotopes (εNd)^19,20^ and the particulate ratio of ^231^Pa to ^230^Th (Pa/Th)^21^. To eliminate structural model biases, we evaluate only the proxy anomalies between the LGM and pre-industrial. We are thus able to obtain a proxy-constrained and spatially complete view of the glacial Atlantic Ocean that also serves as a reference to deglacial perturbations of the AMOC.

**Extended Data Fig. 1:**
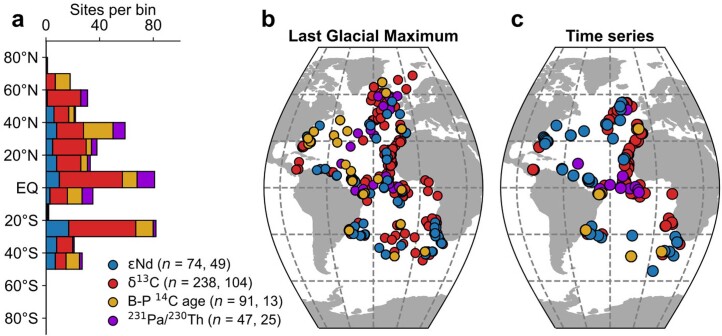
Locations of proxy sites. **a**, Latitudinal distribution of LGM time slice sites binned at 10° intervals. Numbers in brackets indicate number of proxy data for the LGM and deglacial, respectively. **b**, Site locations of proxy records of εNd, δ^13^C, benthic minus planktic (B-P) ^14^C ages, and Pa/Th. **c**, Site locations for time-series records. Note that for the B-P age sites with multiple data points were also counted multiple times.

**Extended Data Fig. 2:**
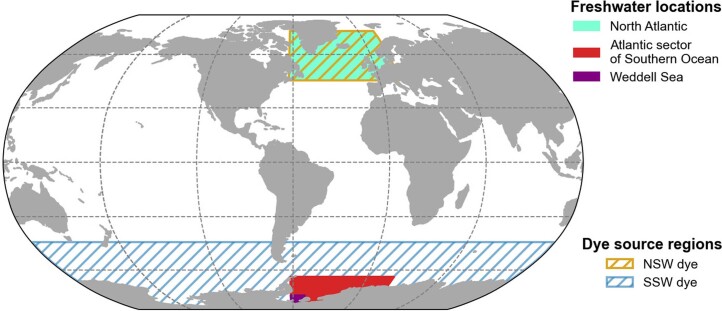
Global map with freshwater and dye tracer regions. Solid boxes mark regions where freshwater is added or removed to generate different LGM AMOC states or where freshwater is added as hosing during the deglaciation as described in Text S1. Dashed regions indicate where the respective dye tracer concentrations are restored to 1 at the surface (that is, the source regions of the dye tracers).

**Extended Data Fig. 3:**
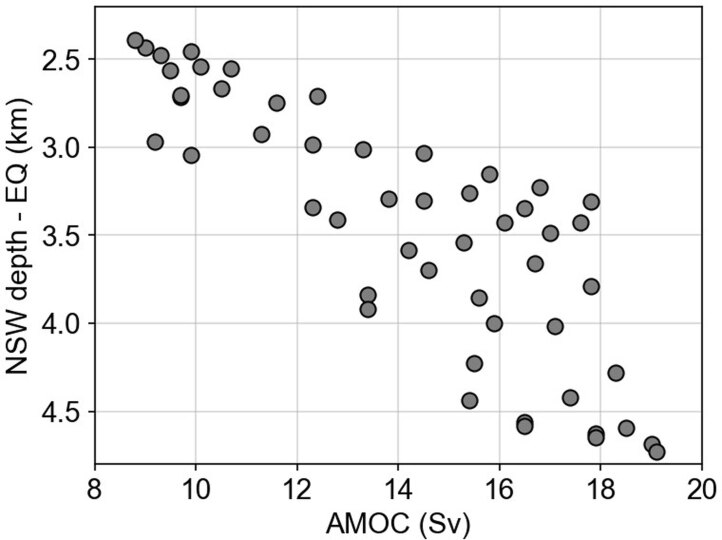
AMOC strength versus northern-sourced water. NSW depth at the equator as determined by the zonally averaged depth of the 50% isoline of 51 LGM simulations exhibiting different combinations of water mass geometry and AMOC strength.

For the LGM, we compiled proxy reconstructions from 18 to 22 ka, providing us with 74 εNd, 238 δ^13^C, 91 B-P ages and 47 Pa/Th data points (Extended Data Fig. 1). The AMOC strength in the 51 LGM simulations spans a range of about 8–19 Sverdrups (Sv; 1 Sv = 10^6^ m^3^ s^–1^) while the depth of northern-sourced water (NSW), defined as the 50% isoline of a North Atlantic dye tracer at the Equator, extends between 2.4 and 4.7 km (Extended Data Figs. 2 and 3 and Methods). To evaluate the different ocean LGM states, we compute the normalized mean absolute errors (MAE, normalized to the range 0 to 1) between reconstructions and simulated proxy distributions weighted by grid-cell volume and measurement uncertainty (where available).

**Fig. 1:**
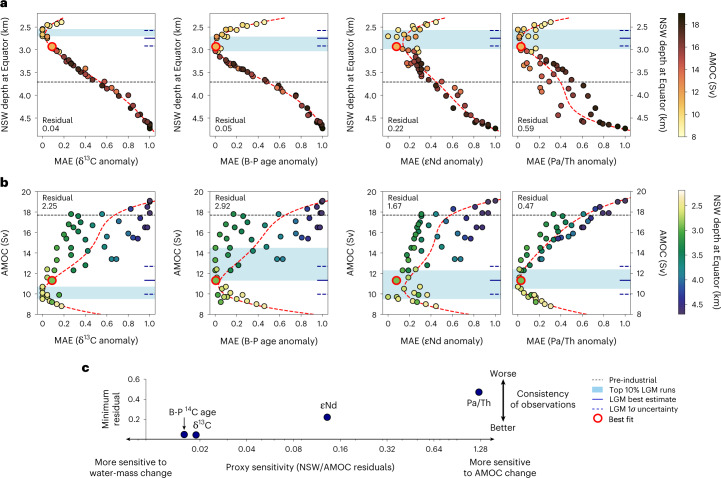
Multi-proxy constraints on the LGM. **a**, Normalized MAE of the 50% isoline of the artificial dye added to the North Atlantic (40–75° N). Normalized MAE for the four proxies and isoline depth is evaluated for 51 LGM simulations. The isoline depth is evaluated at the Equator and below 2 km depth. **b**, Same as **a** but cost function plotted versus the AMOC strength. Black dashed lines indicate the depth of NSW and AMOC at pre-industrial as reference. Blue shadings mark the range of the top 10% best-performing runs with the minimal cost functions for the LGM, that is, the simulations that best fit the proxy reconstructions. Red dashed lines depict polynomial fits (fourth order) of the data. The sum of squared fit residuals for normalized MAE is noted in each panel in normalized units. Solid and dashed short blue lines at the right axis indicate the LGM best estimate from all tracers combined (average of top 10% runs of all proxies weighted by respective fit residual) and its 1*σ* standard deviation, respectively. **c**, Ratios of the fit residuals of NSW over AMOC that indicate the sensitivity of each proxy to either water mass provenance or AMOC change. The *y* axis denotes the minimum residual of NSW depth and AMOC for each proxy and indicates the general consistency of the proxy reconstructions. Note the logarithmic *x* axis.

The resulting correlations between cost functions and AMOC variables paint the first coherent picture of the glacial AMOC state (Fig. 1) as they all exhibit a clear minimum indicative of the best fit between simulations and reconstructions. We further exploit the scatter in the correlations determined by the residuals of polynomial fits to assess how well each proxy traces either water mass mixing or overturning strength. It is worthy of note, however, that this scatter is determined by the proxy’s ability to trace the respective circulation characteristic, the consistency and quality of reconstructions and potentially an incomplete description of the proxy behaviour in the model. This analysis shows that δ^13^C and the B-P radiocarbon age indicate water mass mixing exceptionally well. The latter is commonly used as a proxy for deep ocean ventilation^22,23^, but as noted recently^24^, the difference in radiocarbon ages between NSW and southern-sourced water (SSW) is far greater than any change induced by weakened deep water formation. Here, εNd exhibit only the third-best performance as a tracer of water mass mixing despite being earlier considered a faithful semi-conservative tracer^25^. However, recent studies demonstrated that non-conservative effects play a larger role than previously assumed^20,26^. By contrast, all proxies show only limited ability to trace overturning strength, with Pa/Th exhibiting the best performance regardless of having the smallest database.

**Fig. 2:**
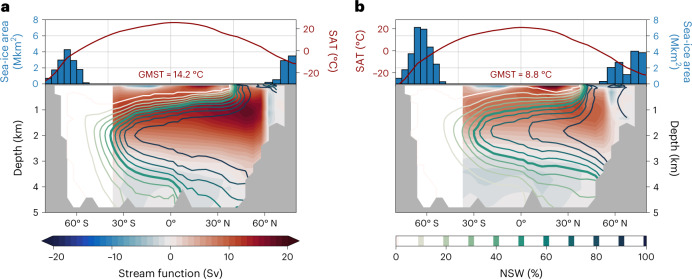
Model climate and ocean states. **a**,**b**, Pre-industrial (**a**) and LGM (**b**) zonally averaged surface atmospheric temperature (SAT) and annual mean sea-ice area (in million km^2^) (top). The global mean surface air temperature (GMST) is also indicated. Filled contours (bottom) show the Atlantic meridional stream function, where positive values indicate clockwise overturning and negative values indicate anticlockwise overturning. Contour lines depict the zonally averaged fraction of NSW in the Atlantic as derived from a North Atlantic dye tracer (45–70° N), normalized to 0% and 100% at 62° S and 3.1 km and at 52° N and 2 km, respectively. Bold lines indicate the 50% isoline.

In summary, all proxies coherently indicate a shoaling of NSW at the LGM by 950 ± 180 m (1*σ*, here and following) relative to the pre-industrial, which corresponds globally to a reduction of NSW from about 28% to 15% (and 53% to 34% in the Atlantic). Interestingly, this holds true even for the previously conflicting δ^13^C and εNd (ref. ^27^), which we find to be a result of non-conservative effects impacting the marine Nd cycle obfuscating the true change in water mass mixing^20^. At the same time, we find that the global proxy distributions can be reconciled only under an ocean state characterized by a relatively weak AMOC with the best fit indicating a circulation strength reduced by 36 ± 8% (6.3 ± 1.4 Sv) relative to the pre-industrial (Fig. 2). Although we cannot exclude the possibility that the exact magnitudes of these changes are to some degree model dependent, we can safely exclude any scenario of stronger than modern^28^ or even only slightly reduced glacial Atlantic overturning^29^. The multi-proxy-constrained LGM circulation with a weaker and shallower AMOC is similar to that inferred from earlier δ^13^C-enabled simulations^24,30–32^ but is here also confirmed by the other proxies, reconciling apparently conflicting proxy evidence.

## Persistent AMOC during HS1

To gain further insight into the nature of AMOC changes during the last deglaciation, we simulated the past 20 kyr with an ensemble of 72 differently evolving overturning circulations forced in addition to our standard deglacial forcing by different freshwater hosing scenarios (Methods). The AMOC responses range from virtually no weakening to full collapses during HS1 and/or YD. Reconstructions and simulated proxy evolutions were binned into 0.5 kyr intervals, and anomalies relative to the LGM were determined for the model–data comparison. Since reconstructed time series are influenced by post-depositional signal attenuation (for example, by bioturbation), we apply Gaussian kernel smoothing to the simulated proxy time series (Methods).

**Fig. 3:**
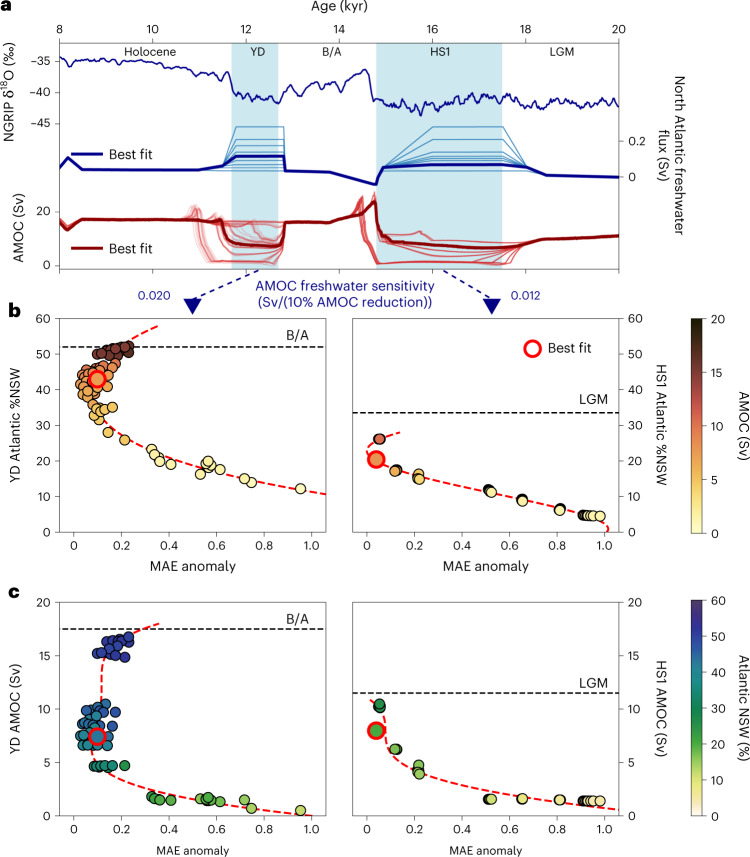
Deglacial constraints on the AMOC. **a**, Time series of Greenland δ^18^O of North Greenland Ice Core Project (NGRIP)^50^, North Atlantic freshwater-flux evolutions of the different transient deglacial simulation (modified after ref. ^31^) and AMOC strength of all deglacial simulations. Thick lines mark the run that provides the best fit between model and reconstructed proxy data. AMOC sensitivity to North Atlantic freshwater hosing is noted below as Sv freshwater required to reduce the AMOC by 10% (see Extended Data Fig. 4). **b**, Combined normalized MAE of all proxies (weighted by LGM residuals (Fig. 1c) and number of records, see Methods for details) versus the fraction of NSW in the Atlantic during the YD and during HS1. **c**, Same as **b** but versus the minimum AMOC strength during the YD and during HS1. Black dashed lines mark NSW fraction and AMOC strength preceding the cold events (that is, LGM for HS1 and B/A for YD). Red dashed lines are polynomial fits and are plotted only as visual aid.

**Fig. 4:**
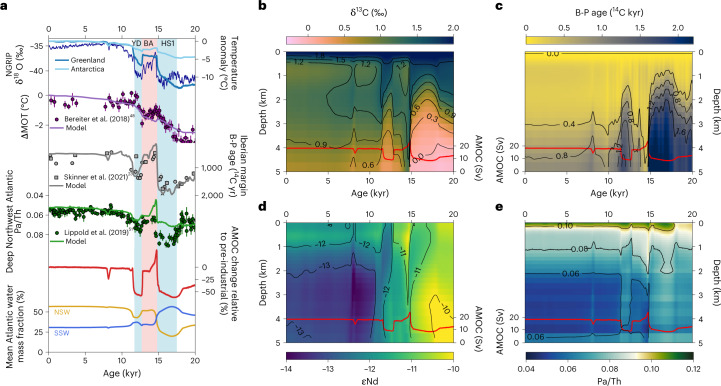
Temporal proxy evolution. **a**, Time series of the past 20 kyr as simulated in the Bern3D model run that best fits the proxy reconstructions as marked in Fig. 3 compared with reconstructions of Greenland δ^18^O (ref. ^50^), MOT^48^ with 1*σ* error bars, B-P from the Iberian margin^23^ with 1*σ* error bars and Pa/Th from the deep Northwest Atlantic^51^ with 2*σ* error bars. Bottom two panels show the simulated AMOC strength relative to the pre-industrial and the fractions of NSW and SSW in the Atlantic (see Extended Data Fig. 2 for source regions). **b**–**e**, Hovmoller plots of δ^13^C (**b**), B-P age (**c**), εNd (**d**) and Pa/Th (**e**) for the zonal averaged North Atlantic at 32° N of the best-fit simulation (see Extended Data Fig. 5 for South Atlantic). Red lines depict the evolution of the AMOC strength.

Contrary to the predominant view inferred from previous geochemical evidence suggesting that the AMOC came to a complete halt during the early deglaciation^5^ due to large North Atlantic meltwater fluxes, we find that a strongly weakened AMOC is unable to reproduce proxy reconstructions of HS1 (Fig. 3 right). Instead, an AMOC with only somewhat reduced vigour relative to the LGM (reduction of about 30%) provides a considerably better representation of reconstructed time series. For example, the large changes in water mass provenance accompanying a strong decrease in overturning strength produce large negative excursions in δ^13^C (<−0.5‰) and very old B-P ages (>2,500 yr) in the North Atlantic that are not reconstructed anywhere to such an extent, and time series are instead characterized by more gradual transitions from glacial to modern values^11,33^. Evidence for little change in water mass provenance also comes from εNd, reconstructions of which also exhibit only moderate changes from the LGM to HS1^26,34^. The notion of a full AMOC collapse was previously underscored by a Pa/Th record of the deep Northwest Atlantic^5^, yet the same magnitude of the HS1 excursion did not emerge as a fully coherent pattern in Pa/Th records throughout the Atlantic, with most time series indicating smaller changes from the LGM to HS1^12,35–37^ as suggested here by our multi-proxy approach. As such, local particle effects could have influenced Pa/Th in the Northwest Atlantic^36,38^, which has major ramifications on the Bermuda Rise time series, which is often considered as a benchmark AMOC record. Our assessment therefore implies that throughout the early deglaciation, SSW occupied a larger fraction of the Atlantic than did water ventilated from the north, which however was never fully replaced as its Atlantic fraction never dropped below 20%. At the very end of HS1, the AMOC then recovered, and the deep circulation switched abruptly to a regime dominated by NSW (Fig. 4a). The relatively small changes from the LGM to the HS1 in AMOC overturning and NSW volume are therefore in conflict with the proposal that an AMOC collapse triggered the early deglacial rise in atmospheric CO_2_^39^ and instead favour mechanisms associated with the Southern Ocean/Pacific^18,40^.

In general, our ensemble of different overturning circulations indicates that small meltwater fluxes are sufficient to substantially perturb the AMOC during the early deglaciation with an average reduction of overturning strength by 10% per 0.012 Sv of freshwater hosing (that is, a full collapse can be achieved with <0.2 Sv; Fig. 4a and Supplementary Fig. 10). Such small fluxes are in general agreement with meltwater-flux reconstructions from sea-level rise that suggest accelerated disintegration of the continental ice sheets during only the latter half of the deglaciation^41,42^. This pronounced sensitivity to meltwater fluxes is the result of an already sluggish circulation preceding HS1, which is close to its tipping point (ref. ^43^).

## Atlantic overturning during the YD

For the YD, the cost functions of the deglacial ensemble express a distinct trend for the fraction of NSW in the Atlantic, while the AMOC strength is somewhat less constrained by the proxy reconstructions (Fig. 3b,c). The model–data comparison indicates that the YD overturning strength was reduced to a similar level as for HS1 (~8 Sv), yet relative to the preceding Bølling–Allerød (B/A), this relates to a reduction of ~60% while the decline for HS1 relative to the LGM amounts to only ~30%. At the same time, the model–data comparison favours high Atlantic fractions of NSW (>30%) close to the level of the B/A, yet these can be realized even with short but pronounced weakening of the AMOC. This ‘decoupling’ of Atlantic water mass provenance from overturning circulation is the result of the short duration of the YD. While the AMOC strength responds directly to buoyancy changes at the North Atlantic deep water formation zones, it takes a few centuries to fully replace NSW by SSW that is only sluggishly advected northward (Fig. 4a). This inertia in replacing water masses indicates that even with high-resolution proxy records, it is challenging to reconstruct past water mass mixing on (sub-)millennial timescales. Moreover, sedimentary disturbances such as bioturbation introduce additional attenuation of proxy time-series excursions, particularly for short events, increasing the difficulty of extracting the true signal amplitude. This explains why constraints on the YD ocean state remained inconclusive for so long.

The freshwater fluxes required to weaken the AMOC to the level of ~8 Sv are substantially different for HS1 and the YD due to the gradually changing climatic and oceanic background conditions in the course of the deglaciation. The YD follows the warm B/A, which is characterized by a vigorous and stable AMOC farther from its tipping point to collapse than the weak and shallow AMOC of the peak glacial. Thus, the overturning is less prone to destabilization during the YD and requires an ~70% higher freshwater flux to achieve a weakening to the same level as HS1; that is, it is about 1.6 times less sensitive. This finding is therefore in structural agreement with the higher meltwater fluxes reconstructed for the second half of the deglaciation^42^ apparently not causing stronger AMOC perturbations^5,23^ as well as its muted response to the final disintegration of the northern hemispheric ice sheets during the early Holocene^44^. Finally, the meltwater flux caused by anthropogenic warming at the end of the twenty-first century^44–46^ is estimated to be in the same order as applied here during the YD. Yet, although the AMOC appears to be more stable during warm climates^47^ at least with regard to meltwater fluxes, the stability of the AMOC during the twenty-first century remains difficult to assess.

## Implications for past mean ocean temperature

**Fig. 5:**
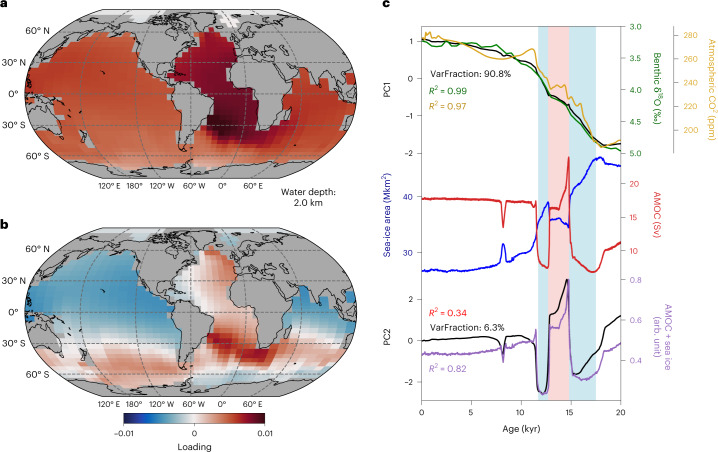
Empirical orthogonal function analysis of ocean temperature. **a**,**b**, Primary (**a**) and secondary (**b**) empirical orthogonal function analyses of the four-dimensional ocean temperature field here as illustration shown for a water depth of 2 km. **c**, Comparisons between the principal components in normalized units and the time-series evolution of ocean and climate variables. From top: PC1 (black) set side by side with the atmospheric CO_2_ concentration^52^ (gold) and a global benthic oxygen isotope stack^53^ (green) that is used in the model to describe ice albedo and aerosol forcing; AMOC (red) and total global sea-ice area (blue); and the PC2 (black) versus combined AMOC and sea-ice variations in arbitrary units (purple; average of the normalized time series of both, with AMOC weighted twice as strong as sea ice). Blue, pink, blue vertical shadings highlight time periods of HS1, B/A and YD, respectively. VarFraction, fraction of total variance.

The newly constrained deglacial AMOC history allows us to assess additional key aspects of the ocean’s evolution such as the mean ocean temperature (MOT), which has been reconstructed from noble gas concentrations in Antarctic ice cores^48^ (Fig. 4a). The comparison reveals that, most strikingly, simulated MOT rather gradually increases throughout the deglaciation and lacks the marked and persistent cooling during the B/A that has previously been interpreted to originate from the bipolar seesaw of millennial-scale AMOC variability^48^. To gain further insight into the dominant processes of MOT variation in the model, we decompose the ocean temperature field into spatio-temporal modes of variability employing empirical orthogonal function analysis (Methods). The first principal component (PC1) explains 91% of the total ocean temperature covariance, and the spatial mode exhibits positive loading across all oceans, with elevated values in the Atlantic (Fig. 5a). This component is therefore clearly associated with the gradual increase in radiative forcing of greenhouse gases, ice albedo and aerosols during the deglaciation, which can explain more than 97% of the variation, and echoes assessments of the surface oceanic and atmospheric temperature evolution over the past 22 kyr (refs. ^6,49^). We interpret the higher loading of the Atlantic as the steady expansion of NSW, which concurrently warmed more strongly than SSW during the deglaciation.

**Extended Data Fig. 6:**
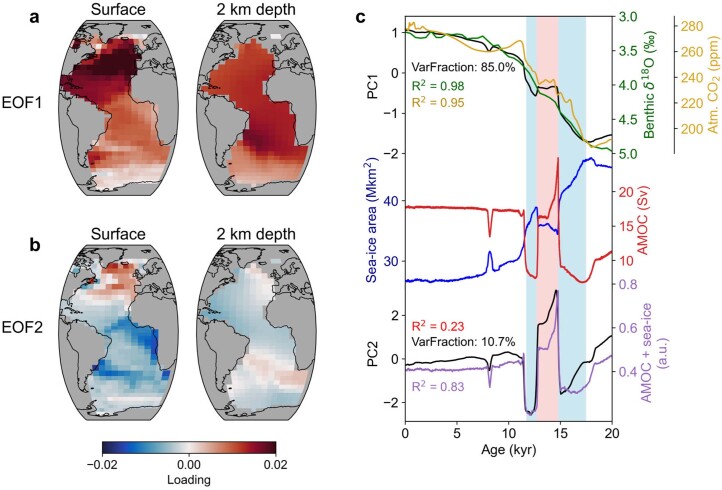
Temperature EOF analysis for the Atlantic only. Spatial loading distributions of **a**, EOF1 and **b**, EOF2 for (left) the surface ocean averaged over the top 126 m and (right) at 2 km water depth. **c**, First and second principal components in comparison to (top) benthic δ18O (green, ref. ^49^), atmospheric CO2 concentration (golden, ref. ^53^), and (mid, bottom) AMOC strength (red), total sea-ice area (blue), and combined AMOC and sea-ice variations in arbitrary units (purple; average of the normalized timeseries of both, with AMOC weighted twice as strong as sea-ice). Blue and pink vertical shadings highlight time periods of Heinrich Stadial 1 and Younger Dryas and Bølling-Allerød, respectively.

The second principal component (PC2) describes only about 6% of the total ocean temperature variation but displays pronounced millennial-scale variability and a spatial dipole pattern with positive loading in the Atlantic and Southern Ocean and negative loading in the Pacific and Indian Ocean at 2 km water depth (Fig. 5b,c) and is thus deemed physically interpretable. The AMOC evolution shares many features with the time series of PC2 but is unable to fully explain it (*R*^2^ = 0.34). An additional trend is apparent that matches the decline in sea-ice extent during the deglaciation, which can be understood by the direct link between sea-ice formation/melt and ocean heat transport by the AMOC. Combined, the AMOC and sea-ice evolution can explain 86% of PC2. We hence interpret the dipole loading pattern as resulting from the bipolar seesaw caused by the AMOC (see Extended data Fig. 6 for classical north–south dipole pattern at the surface). As such, in contrast to the hypothesis that the bipolar seesaw might be responsible for the deglacial variations in reconstructed MOT^48^, we here find that the effect on MOT appears negligible because its contribution to global temperature variance is dwarfed by the radiative forcing in addition to smaller regional warming and cooling trends, roughly cancelling each other out. Although this finding might be model dependent, it reveals an urgent need to assess alternative processes that may have caused multi-century-scale mean ocean cooling independent of radiative forcing during the last deglaciation.

## Methods

### Model description

The Bern3D Earth system model of intermediate complexity (v.2.0) consists of a dynamic geostrophic–frictional balance ocean^54,55^ coupled to a thermodynamic sea-ice component and a single-layer energy–moisture balance atmosphere^56^. All model components have a spatial resolution of 41 × 40 grid cells with the ocean having 32 logarithmically scaled depth layers. The physical ocean features an isopycnal diffusion scheme and a Gent–McWilliams parameterization for eddy-induced transport^57^. Wind stress and cloud cover are prescribed as monthly climatologies^58^.

The prognostic biogeochemistry module calculates export production and remineralization in the water column of dissolved and particulate organic matter (POM), calcium carbonate (CaCO_3_) and biogenic opal^18,59,60^. Carbon chemistry and air–sea gas transfer parameterizations follow the Ocean Carbon-Cycle Model Intercomparison Project Phase 2 (OCMIP-2) protocols with updated Schmidt number calculations^61^ and carbon chemistry^62^. Diverging from the OCMIP-2 protocol, the gas transfer velocity scales linearly with wind speed^63^.

The four ocean tracers that are the focus of this study—Pa/Th, Nd isotopes, stable carbon isotopes and radiocarbon—are all simulated explicitly in the Bern3D model. Radiocarbon is implemented following the OCMIP-2 protocol, and stable carbon isotopes are subject to fractionation during air–sea gas exchange, in the marine chemical carbonate equilibrium system and during photosynthesis^18^. Carbon isotope fluxes are further simulated between the land–atmosphere and ocean–atmosphere interfaces. Previously, simulated δ^13^C of dissolved inorganic carbon (δ^13^C_DIC_) displayed an offset of about 0.4‰ compared with observations^64^. We therefore tuned δ^13^C_DIC_ by varying the fractionation coefficients of the marine carbonate system and photosynthesis and the lifetime of dissolved organic matter within their uncertainties. While the tuning eliminated the global offset of 0.4‰, model–data discrepancies remain in the intermediate North Pacific and the deep North Atlantic. Both, however, are related to more fundamental model deficits in simulating ocean physics in these regions. The Nd cycle has been fully implemented in the Bern3D model^19,65^, which includes the three sources of dust, rivers and marine sediments and the internal cycling and removal by reversible scavenging. The implementation has recently been further revised with new constraints of observations and evaluated for the LGM and deglaciation^20^. Pa and Th are simulated following ref. ^21^, which includes explicit tracers of the dissolved and particulate phases for each radionuclide. In contrast to ref. ^21^, we do not employ a parameterization for boundary scavenging and replaced the previously globally homogeneous bottom scavenging with a spatially heterogeneous data-constrained map from benthic nepheloid layers^66^. Finally, we implemented particle concentration-dependent scavenging as observed in the modern ocean^67^ and retuned the scavenging parameters to fit tracer concentrations to the latest seawater observations.

To complement the geochemical tracers, we further simulate ideal age, an explicit tracer subject to advection, diffusion and convection, which increases by 1 yr yr^−1^ everywhere except at the surface ocean, where it is reset to zero. In addition, we simulate two dye tracers, which are restored to a concentration of 100% in the North Atlantic (45–70° N) and Southern Ocean (south of 40° S) surface (Extended Data Fig. 2) and behave fully conservatively in the ocean’s interior; that is, their concentration decreases only by mixing with water masses sourced from outside the respective dye source region. These tracers thus allow for determining the fraction of NSW and SSW (see Supplementary Figs. 2 and 3 for global distributions and their difference from the pre-industrial).

### LGM forcings

Before applying any LGM forcings, we spun up the model over 35 kyr corresponding to boundary conditions of 1765 ce, except a closed Bering Strait, to achieve a fully equilibrated state. The LGM forcings also applied here are described in detail in ref. ^20^. In brief, to achieve a realistic LGM steady state, we start from the pre-industrial equilibrium conditions and run the model for 20 kyr under constant LGM forcing. This forcing includes orbital parameters set to 20 ka^68^ and radiative forcing corresponding to greenhouse gas concentrations of CO_2_ = 191 ppm, CH_4_ = 370 ppb and N_2_O = 208 ppb^69^. While the radiative forcing of CO_2_ is prescribed, its biogeochemistry is determined interactively. To get better agreement with reconstructed atmospheric and oceanic δ^13^C, we add 450 GtC with δ^13^C = −24‰ to the glacial atmosphere at the beginning of the LGM spin-up as suggested by ref. ^64^, representing the lower glacial land carbon storage. Further, the continental ice-sheet extent and the related changes in albedo are set to the reconstructions by ref. ^70^. The corresponding freshwater relocation responsible for the increase in salinity by about 1 practical salinity unit is performed during the first 2 kyr of the LGM spin-up. We additionally apply LGM wind-stress anomalies^71^ and account for the increased vertical mixing due to lower sea-level increasing tidal dissipation as described by refs. ^32,43,72^. To achieve global mean surface temperatures in better agreement with recent model–data assimilation^6^, we apply an additional top-of-the-atmosphere aerosol forcing of −2.5 W m^–2^, which in total leads to a cooling relative to the pre-industrial of −5.4 to −6.1 °C depending on the ocean state. Because biogenic particle remineralization is not fully dynamically implemented in the Bern3D model, we reduce the POM remineralization in the upper water column as a consequence of lower microbial activity due to the lower ocean temperatures during the LGM^73^.

To obtain ocean-circulation states that differ not only in AMOC strength but also in water mass geometry, we continuously apply small freshwater fluxes to the North Atlantic (−0.04 to +0.1 Sv) and/or the Weddell Sea (−0.2 to +0.25 Sv) (see Extended Data Fig. 2 for regions) that are compensated for in the North Pacific and Southern Ocean, respectively. These freshwater fluxes represent hydrological processes not explicitly or only poorly represented in the model as well as continental meltwater fluxes not explicitly accounted for in the model. Here we further extend the combinations and variations of these two freshwater fluxes compared with ref. ^20^ to obtain in total 51 different LGM ocean states. The best-fit scenario comprises a freshwater-flux correction of 0.08 Sv to the North Atlantic and 0.10 Sv to the Weddell Sea.

### Deglacial forcings

The deglacial forcing applied here follows ref. ^20^ and includes prescribed orbital and greenhouse gas forcing, aerosol forcing, albedo changes of the continental ice sheets, wind stress and changes in vertical mixing due to lower sea-level stand. Biogeochemical variables that were prescribed for the LGM (iron dust flux, Nd input fluxes and POM remineralization) are scaled back to their pre-industrial values on the basis of the evolution of the global benthic δ^18^O stack of ref. ^53^ that represents the sea-level evolution and thus also continental ice-sheet extent. The vertical remineralization profile of POM is scaled back on the basis of the combined atmospheric CO_2_ and benthic δ^18^O evolutions (weighted mean with weights of 0.4 and 0.6 for CO_2_ and benthic δ^18^O, respectively, based on the modern contributions to the total radiative forcing^74^). Atmospheric radiocarbon concentrations were prescribed following IntCal20^75^. In addition, in the course of the deglaciation and early Holocene, we remove the 450 GtC that were added to the atmosphere during the LGM spin-up, also assuming δ^13^C = −24‰. From 18 to 11 ka, 200 GtC are removed with a constant rate of ~0.028 GtC yr^–1^. The remaining 250 GtC are then removed over the Holocene following the land biosphere uptake constraints by ref. ^76^. The small freshwater-flux corrections required to achieve the best-fit LGM ocean state were scaled back to zero over the deglaciation on the basis of benthic δ^18^O. To generate the 72 different AMOC evolutions, freshwater hosings of 0.035–0.280 Sv were applied to the North Atlantic during HS1 and/or the YD (45–70° N; turquoise region in Extended Data Fig. 2) that were not compensated for in other regions of the ocean. The maximum freshwater fluxes are chosen to be equivalent to 120 m of sea-level rise over the deglaciation. For runs with lower North Atlantic freshwater hosing, additional freshwater was applied to the Atlantic sector of the Southern Ocean (red region in Extended Data Fig. 2) to achieve roughly the same 120 m of sea-level rise following the same evolution as the North Atlantic hosing^77^. The Bering Strait was opened at 12 ka as suggested by reconstructions of local sea-level rise^78^.

### LGM proxy compilations

Neodymium isotope data were compiled by ref. ^20^ and are based on previous efforts^26^ updated with the newest reconstructions. Stable carbon isotope and radiocarbon datasets by refs. ^24,79^ were further expanded with previously missing and newly published data. Pa/Th data are newly compiled and include only sites for which both well-dated LGM (18–22 ka) and late Holocene (0–6 ka) data were available. All data points in both time intervals were averaged, and the difference was taken to derive the anomalies used for the model–data evaluation. Detailed information on all proxy compilations is documented in Supplementary Tables 1–4, and locations are plotted in Extended Data Fig. 1 as small circles. Absolute and LGM minus late Holocene anomalies are plotted in comparison with the simulated LGM proxy distributions and their difference from the pre-industrial as global transects from the North Pacific to the Southern Ocean to the North Atlantic in Supplementary Figs. 4–7.

### Deglacial proxy compilations

To evaluate the model’s performance in simulating the last deglaciation reconstructed time series of δ^13^C, εNd, B-P age and Pa/Th were compiled. Peterson and Lisiecki^33^ previously collated δ^13^C deglacial time series and provided revised age models based on regional radiocarbon compilations. We here removed duplicate records of this compilation and expanded the database with newly published time series from the Iberian margin by ref. ^23^. Pa/Th records were newly compiled here and include only sites that were dated by radiocarbon and that exhibit at least one data point in the time interval from 18.5 to 22 ka. Neodymium isotope and B-P age records were also newly compiled here with the same constraint to exhibit at least one data point in the range of 18.5 to 22 ka. For εNd, all records from the North Atlantic that exhibit notable overprinting from ice-rafted debris have been excluded (see ref. ^80^). The detailed information on all proxy time-series compilations can be found in Supplementary Tables 5–8.

### Model–data comparison

The model’s skill in simulating proxy distributions in agreement with reconstructions was evaluated by calculating the MAE as a cost function that is to be minimized:

$${\mathrm{MAE}} = \frac{1}{n}\mathop {\sum}\limits_{i = 1}^n {\left| {y_i - x_i} \right|}$$

where *y*_*i*_ and *x*_*i*_ are observed and simulated values of a quantity under consideration. The data have been weighted by the grid-cell volume and, where available, by the measurement uncertainties (εNd, B-P age and Pa/Th). The MAEs for each proxy were then normalized to values between 0 and 1 for better comparability. To eliminate structural model biases, we evaluated the LGM anomaly with respect to modern. Since marine sediment cores often lack a modern core top, we averaged the proxy data for each site over the late Holocene (0–6 ka) to get a modern equivalent. Since the ocean circulation was markedly stable over this period^51^, the additional uncertainty introduced by this should be minimal. Simulated proxy anomalies have been calculated relative to a fully equilibrated pre-industrial control run. Global proxy distributions of the LGM and the difference from the control run are depicted in Supplementary Figs. 4–7, also marking the reconstructions.

For the evaluation of the transient simulations, LGM averages were calculated over the period of 18.5 to 22 ka for the observations and 18.5 to 20 ka for the model runs to be as inclusive as possible with regard to observations while the simulations only start at 20 ka. Beyond that, reconstructions and simulated proxy distributions were binned in 500 yr intervals. An interval of 500 yr was chosen as a compromise between still being able to resolve the millennial-scale variability of the last deglaciation and the temporal resolution of many proxy records, which often do not exceed 500 yr. To take into account bioturbation that leads to post-depositional attenuation of the original oceanic isotope signal in the sediment, we apply Gaussian kernel smoothing to the simulated proxy time series. On the basis of the global average bioturbation length of about 6 cm (ref. ^81^) and an average sedimentation rate of about 10 cm kyr^–1^, we chose a standard deviation of the Gaussian distribution of 600 yr. We then calculated the deviation from the LGM average. The cost function was calculated individually for HS1 and YD for the periods of 15 to 18.5 ka and 9 to 14 ka, respectively, with each site weighted by the total number of bins containing at least one data point. To calculate the final model score displayed in Fig. 3, each proxy was weighted by the number of records available for that proxy and the minimum residual of the LGM simulations (Fig. 1c), which described how well each proxy represents ocean-circulation changes.

### Empirical orthogonal function analysis

We applied empirical orthogonal function analysis to the transiently simulated ocean temperature field of the past 20 kyr to assess the spatio-temporal modes that dominate its variability. For this, we followed standard procedure^82^ and first centred the simulated four-dimensional field to time-mean zero, which was subsequently weighted by the square root of the cosine of the corresponding latitude. The first mode already explains more than 91 ± 5% of the ocean temperature variation since the LGM, followed by the second mode explaining about 6 ± 0.3% (uncertainty determined by the ‘North test’; ref. ^83^). We only deemed these two modes physically interpretable as their principal component time series are distinct from background noise and clearly exhibit variations mirroring physical variables (radiative forcing for PC1 and AMOC plus sea-ice extent for PC2) in their magnitude and timing. Further, despite explaining only 6% of the variation, the second mode exhibits a spatial loading pattern that has previously been identified as a fingerprint of the bipolar seesaw^6,84^ underscoring its physical meaning.

## Online content

Any methods, additional references, Nature Portfolio reporting summaries, source data, extended data, supplementary information, acknowledgements, peer review information; details of author contributions and competing interests; and statements of data and code availability are available at 10.1038/s41561-023-01140-3.

## Supplementary information

Supplementary Information Supplementary Figs. 1–11.

Supplementary Tables 1–8 Data compilations of δ^13^C, B-P age, εNd and Pa/Th for the LGM and last deglaciation.

## Figures and Tables

**Extended Data Fig. 4:**
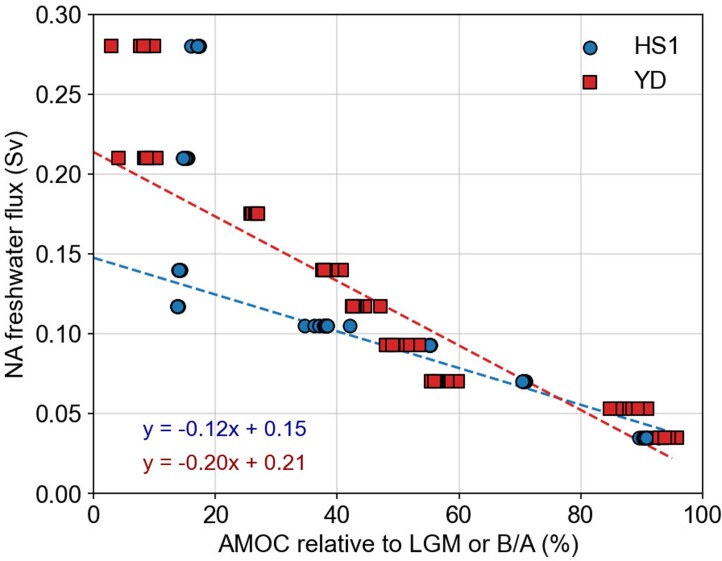
Sensitivity of AMOC to freshwater forcing. AMOC strength at HS1 and YD relative to the previous time periods (that is, LGM for HS1 and B/A for YD) versus the North Atlantic freshwater flux during hosing (see Extended Data Fig. 2 for region where the forcing is applied). Dashed lines mark linear regressions for simulations that do not exhibit already a fully collapsed AMOC state, indicating that 0.012 and 0.020 Sv of freshwater flux are required to reduce the AMOC by 10% during HS1 and YD, respectively.

**Extended Data Fig. 5:**
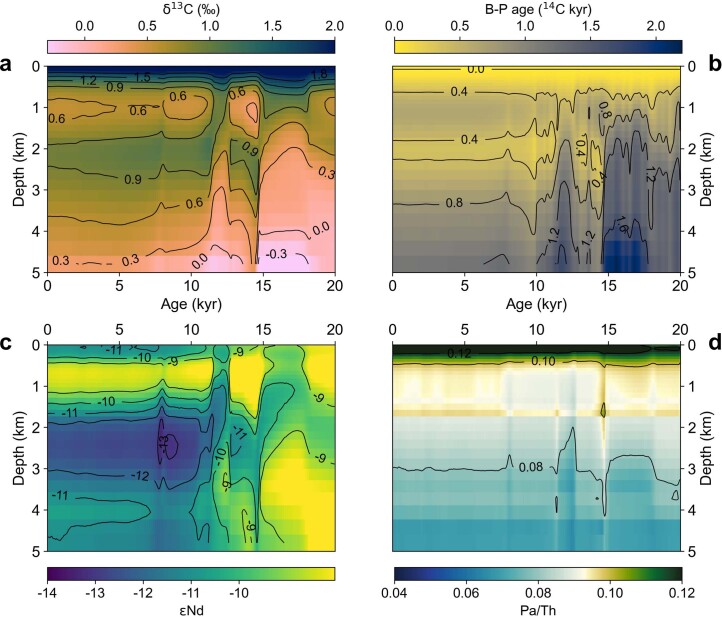
Proxy evolution in the South Atlantic. Hovmoller plots of zonal averages of the South Atlantic (27°S) for **a**, stable carbon isotopes, **b**, benthic minus planktic radiocarbon age, **c**, neodymium isotopes, and **d**, Pa/Th.

## Data Availability

Data compilations of δ^13^C, B-P age, εNd and Pa/Th for the LGM and last deglaciation can be found in the supplementary data and at Zenodo (https://zenodo.org/record/7404303). The Bern3D model output is publicly available via Zenodo (https://zenodo.org/record/7540200).
