# Supplementary material for: Multi-proxy constraints on Atlantic circulation dynamics since the last ice age
Source: Nat Geosci. 2023 Apr 3;16(4):349–56. doi: 10.1038/s41561-023-01140-3 (PMC10089918; doi:10.1038/s41561-023-01140-3)
Supplement: Supplementary file 1 — Supplementary Figs. 1–11. [file 41561_2023_1140_MOESM1_ESM.pdf]

nature geoscience

Article

https://doi.org/10.1038/s41561-023-01140-3

Multi-proxy constraints on Atlantic circulation dynamics since the last ice age

In the format provided by the authors and unedited

Supplementary Information for

Multi-proxy constraints on Atlantic circulation dynamics since the last ice age

Frerk Pöppelmeier et al.

Supplementary Figures S1 to S11

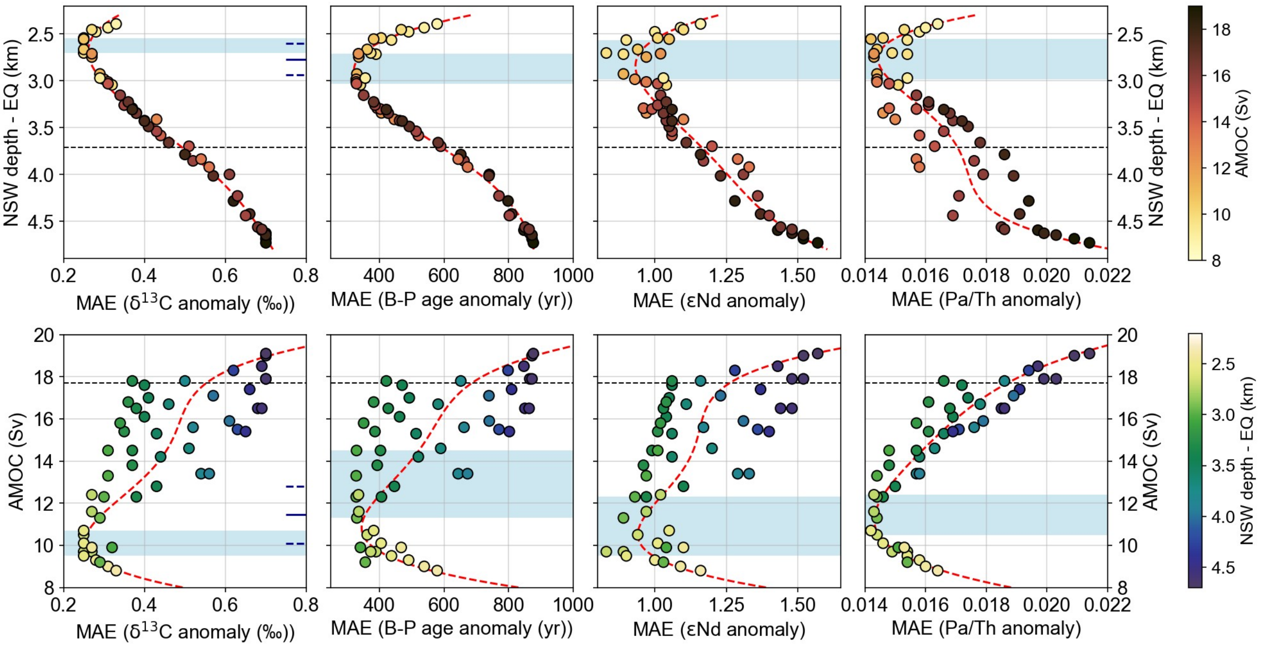

Fig. S1. Same as Fig. 1 but cost functions (MAE) are not normalized. Black dashed lines indicate the depth of NSW and AMOC at pre-industrial as reference. Blue shadings mark the range of the top 10% best performing runs with the minimal cost functions for the LGM, i.e., the simulations that best fit the proxy reconstructions. Red dashed lines depict polynomial fits (forth order) of the data. Solid and dashed blue lines indicate the LGM best estimate from all tracers combined (average of top 10% runs of all proxies weighted by respective fit residual) and its 1σ standard deviation, respectively.

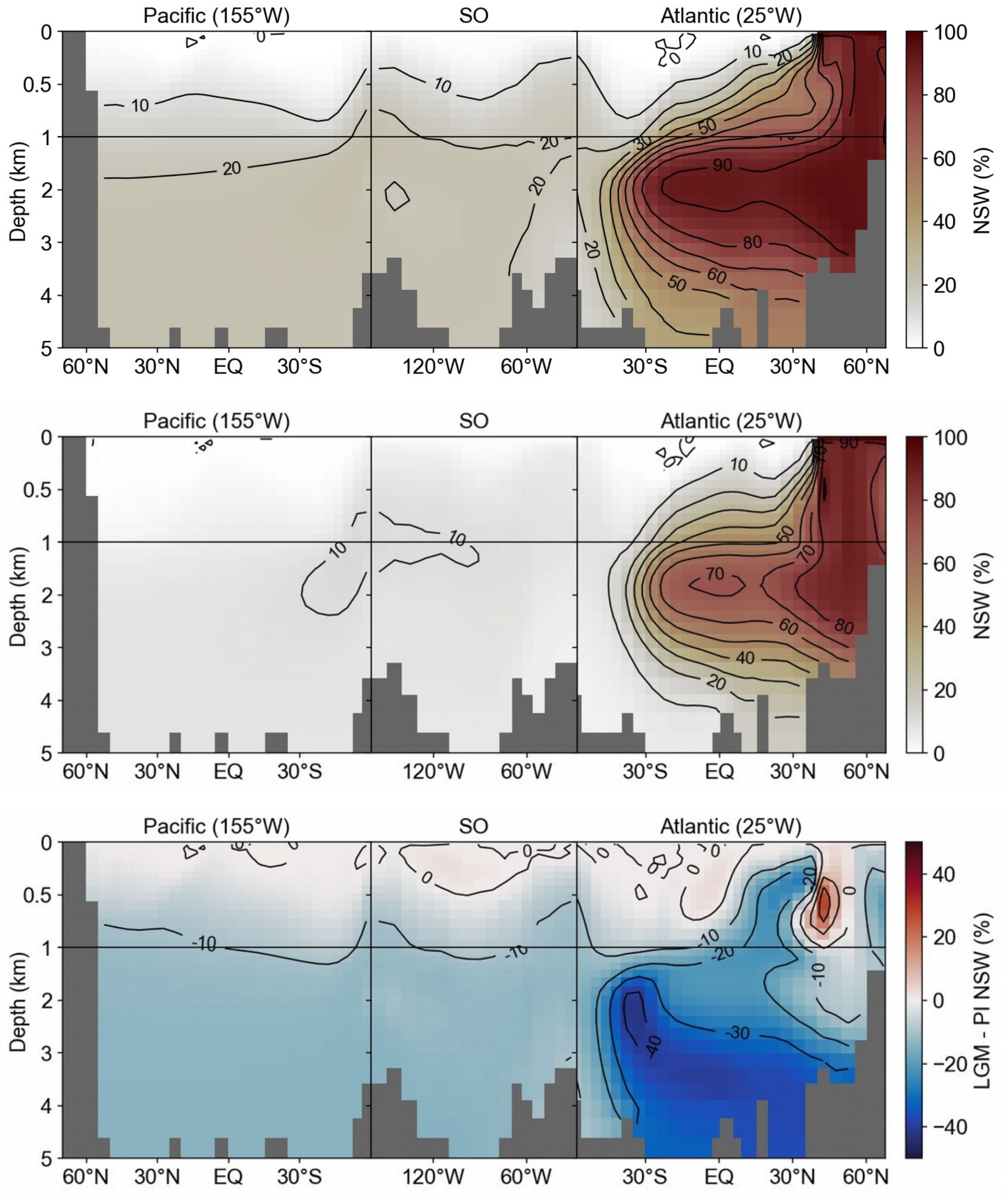

Fig. S2. Global sections from the North Pacific to the Southern Ocean to the North Atlantic of (top) the fraction of northern-sourced water (NSW) for the pre-industrial, (mid) the LGM simulation that best fits the reconstructions (see Fig. 2) and (bottom) the difference of the LGM to the pre-industrial distribution of NSW along the same transect.

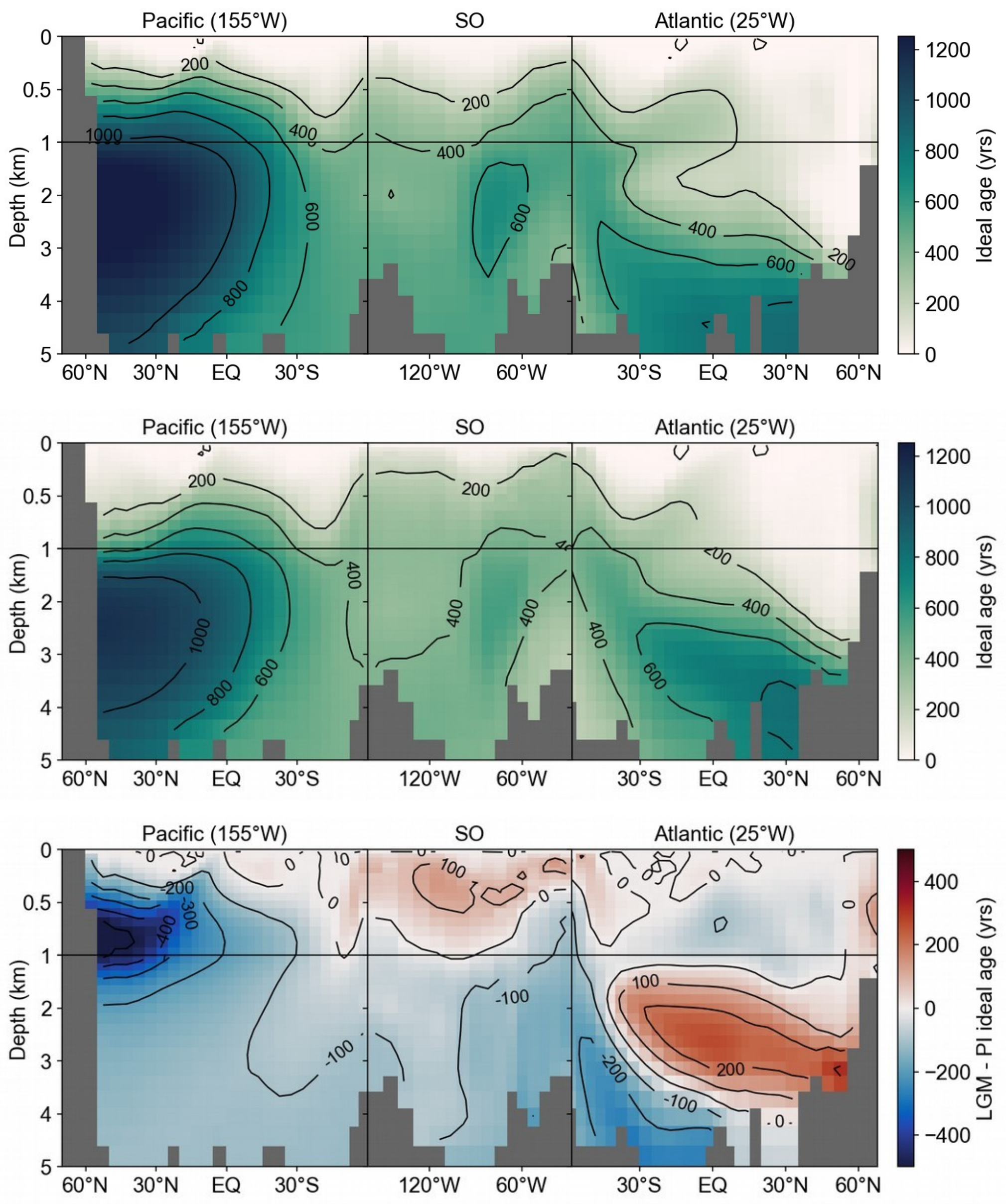

Fig. S3. Global sections from the North Pacific to the Southern Ocean to the North Atlantic of an ideal age tracer that is restored to zero at the sea surface for (top) the pre-industrial, (mid) the LGM simulation that best fits the reconstructions (see Fig. 2), and (bottom) the difference of the LGM to the pre-industrial ideal age distribution along the same transect.

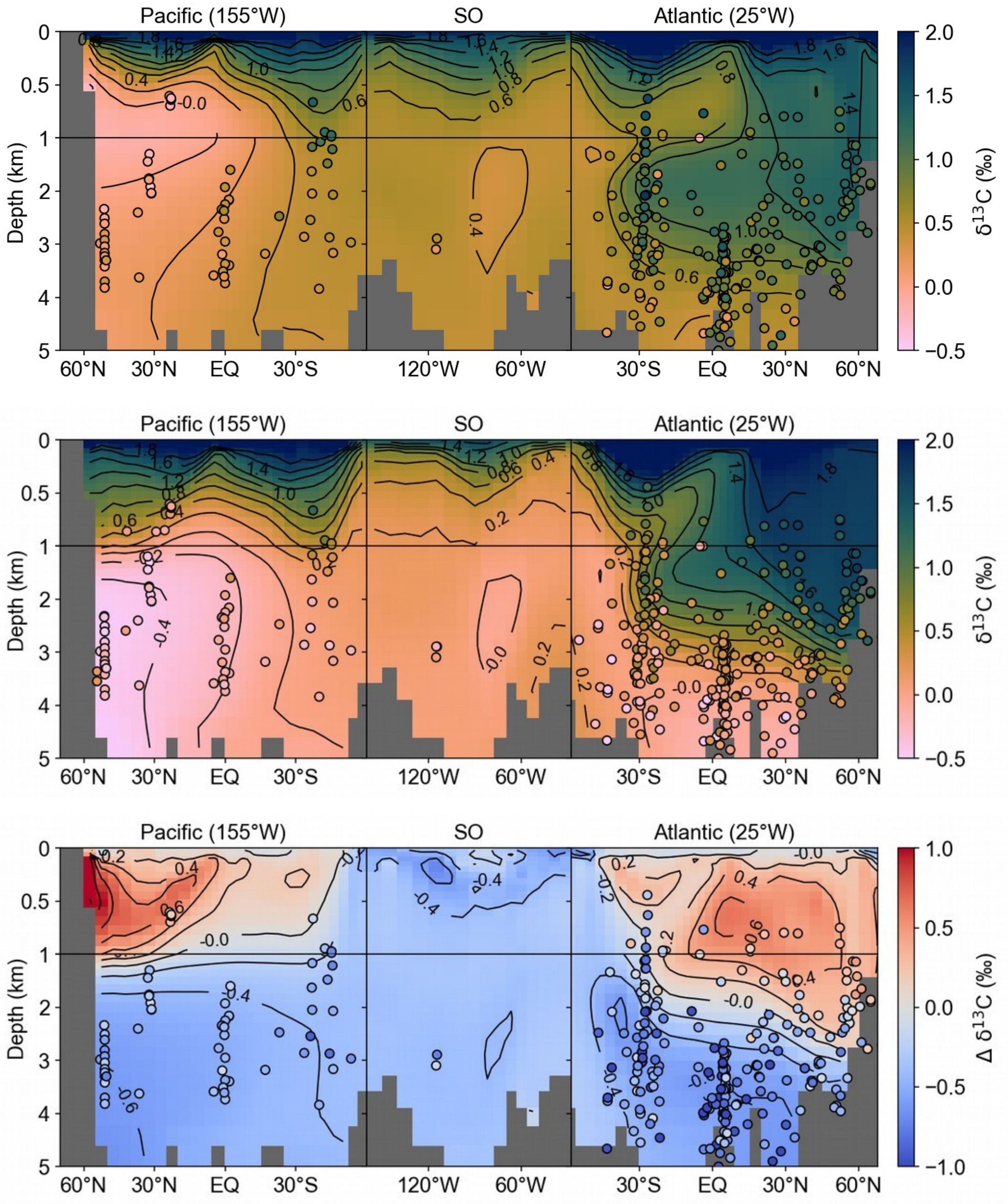

Fig. S4. Global distributions of $\delta^{13}C$ (top) for the pre-industrial, (mid) the LGM, and (bottom) the difference of the LGM compared to the pre-industrial. Filled contours depict the simulated distributions and circles mark the reconstructions on the same color map.

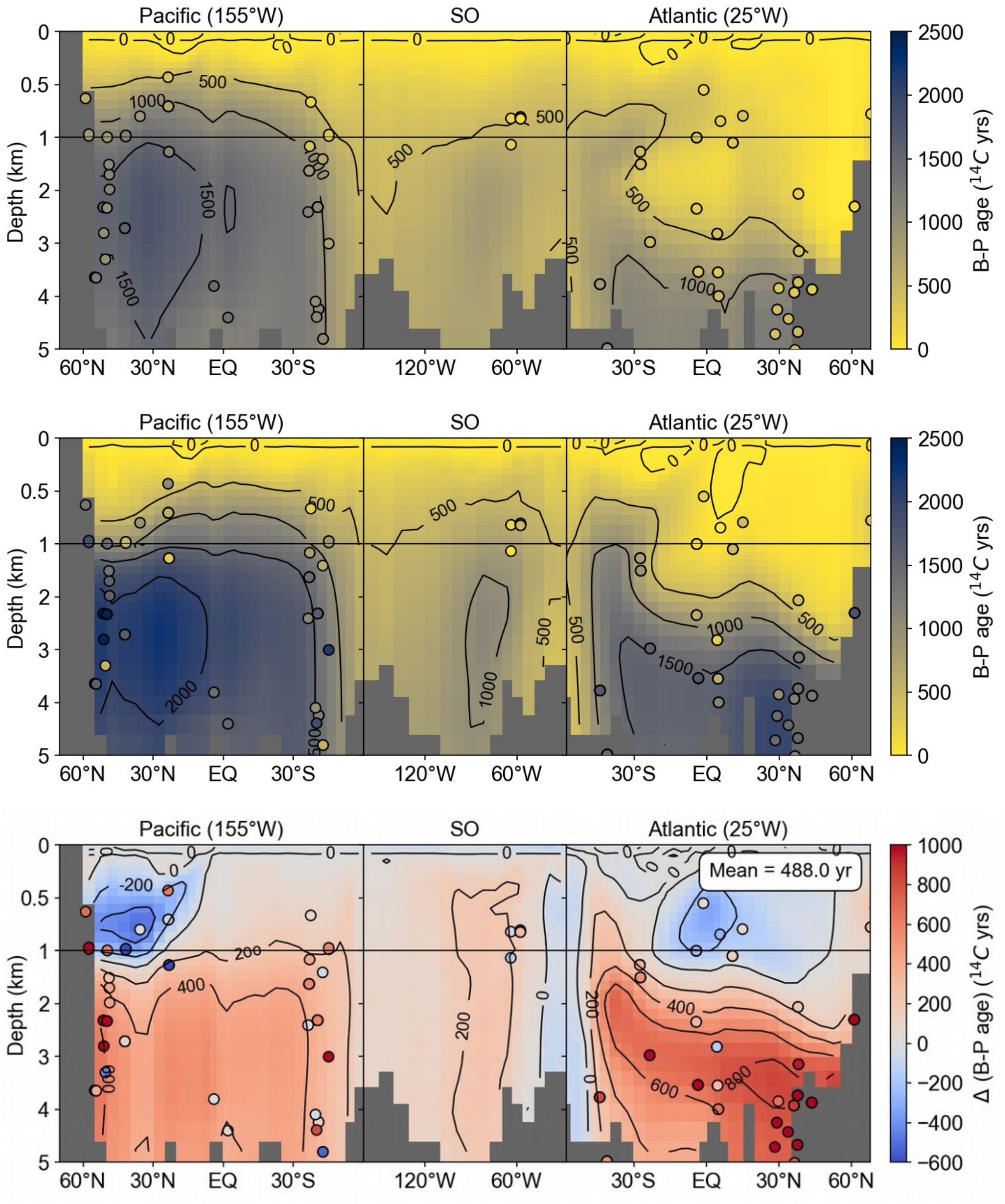

Fig. S5. Global distributions of benthic minus planktic radiocarbon ages (top) for the pre-industrial, (mid) the LGM, and (bottom) the difference of the LGM compared to the pre-industrial. Filled contours depict the simulated distributions and circles mark the reconstructions on the same color map. The global mean LGM-PI difference of the B-P age is 488 years.

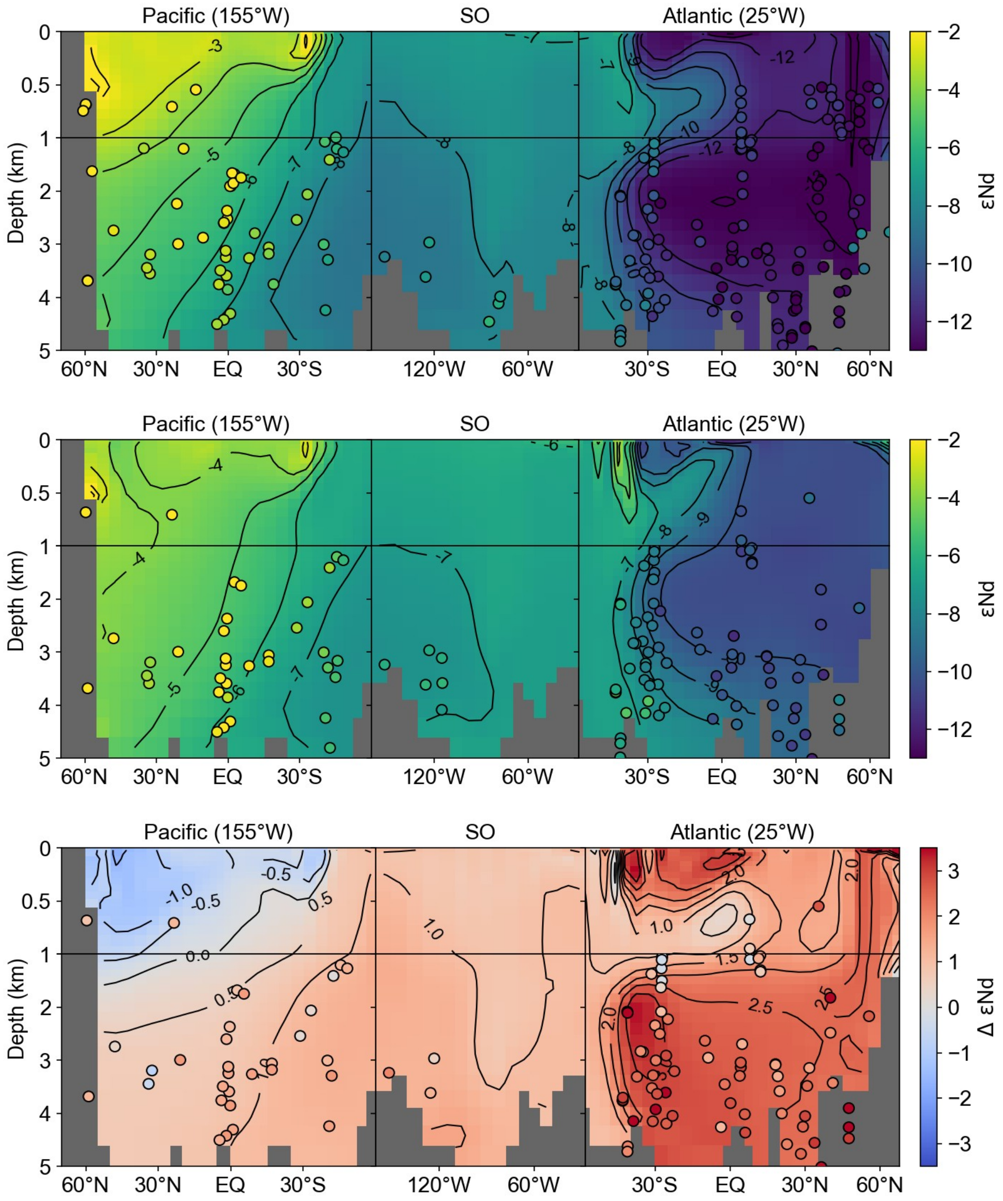

Fig. S6. Global distributions of εNd (top) for the pre-industrial, (mid), the LGM, and (bottom) the difference of the LGM compared to the pre-industrial. Filled contours depict the simulated distributions and circles mark the reconstructions on the same color map.

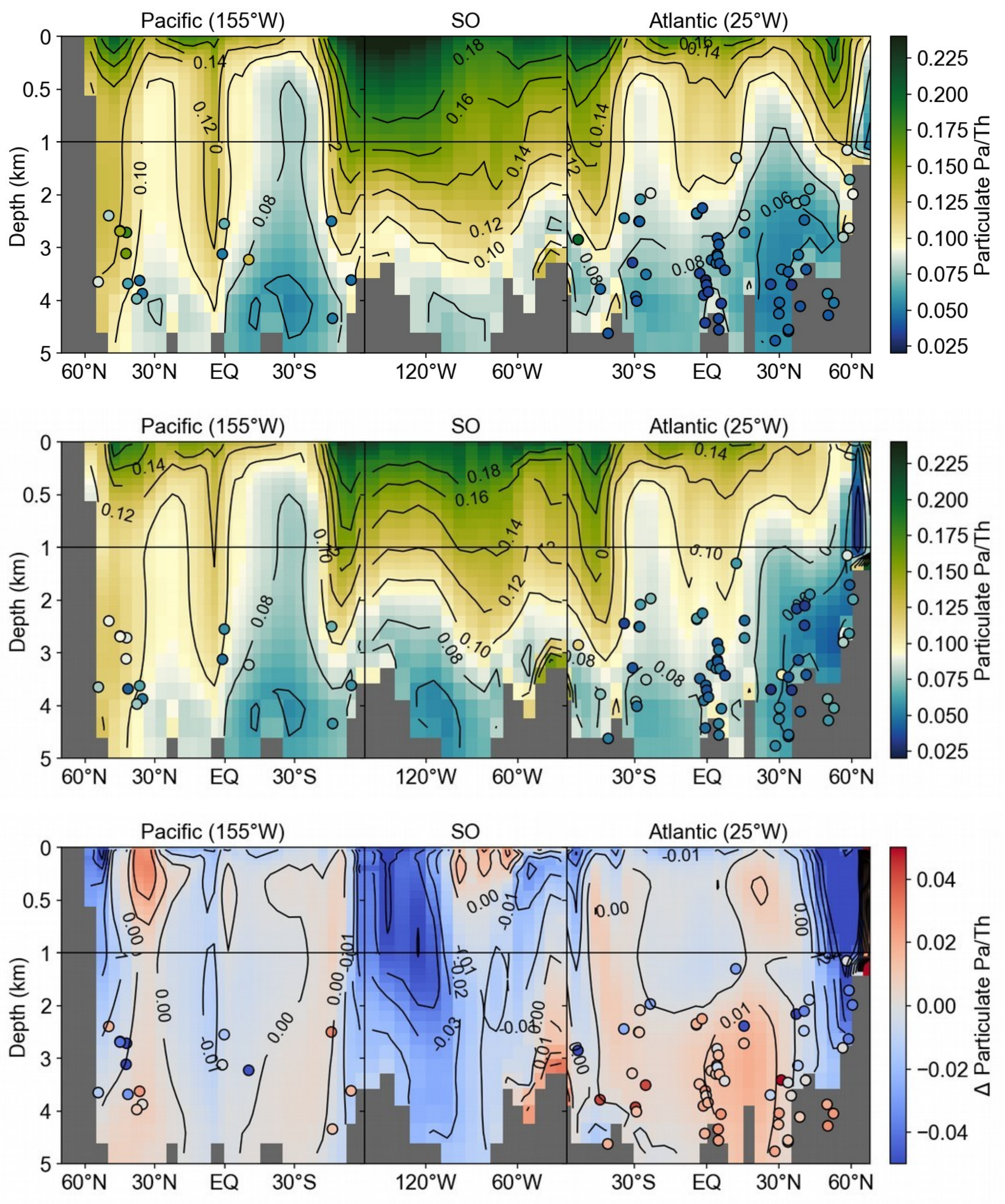

Fig. S7. Global distributions of particulate Pa/Th (top) for the pre-industrial, (mid) the LGM, and (bottom) the difference of the LGM compared to the pre-industrial. Filled contours depict the simulated distributions and circles mark the reconstructions on the same color map.

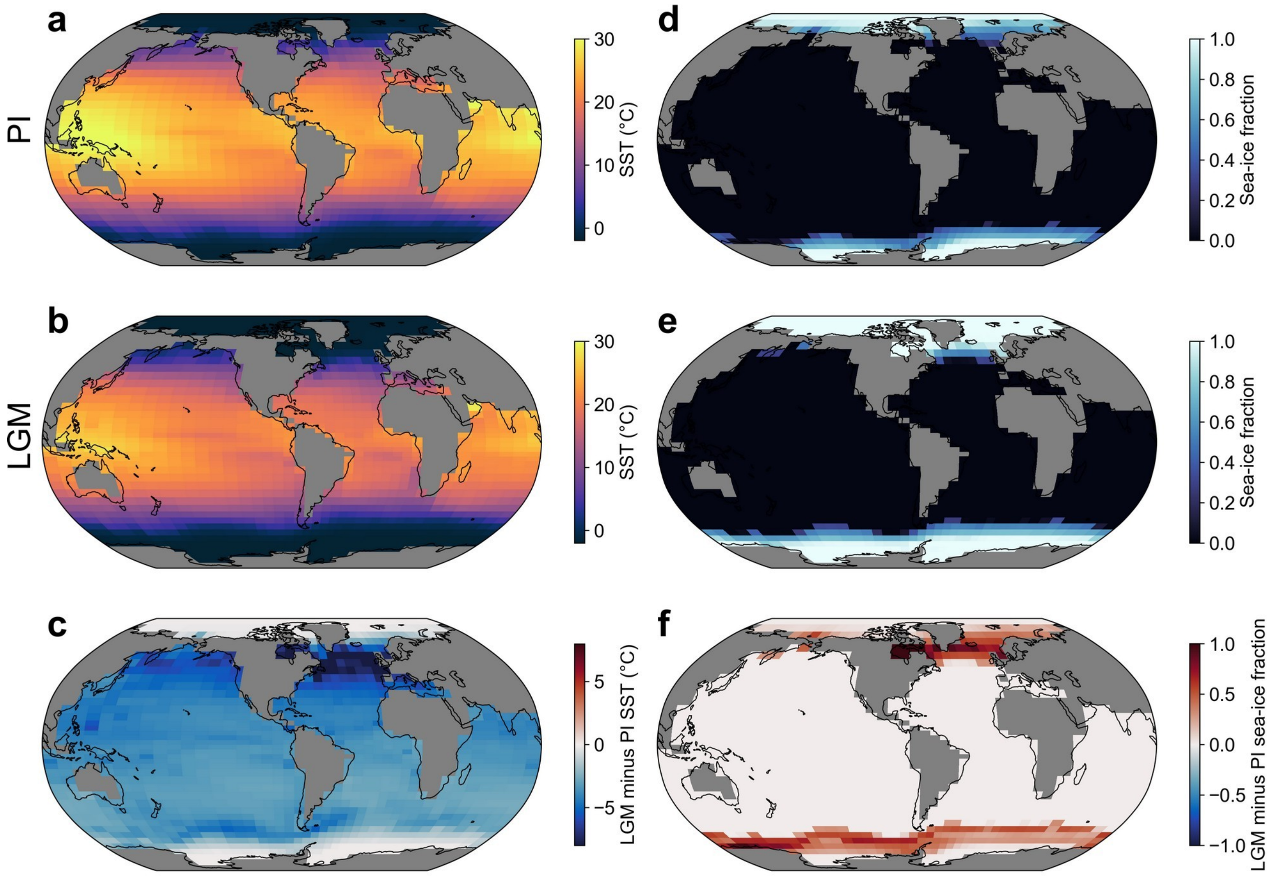

Fig. S8. (*Left*) Sea surface temperature and (*right*) sea-ice fraction of (a,d) the pre-industrial (PI), (b,e) Last Glacial Maximum (LGM) best-fit simulation, and (c,f) the difference between both.

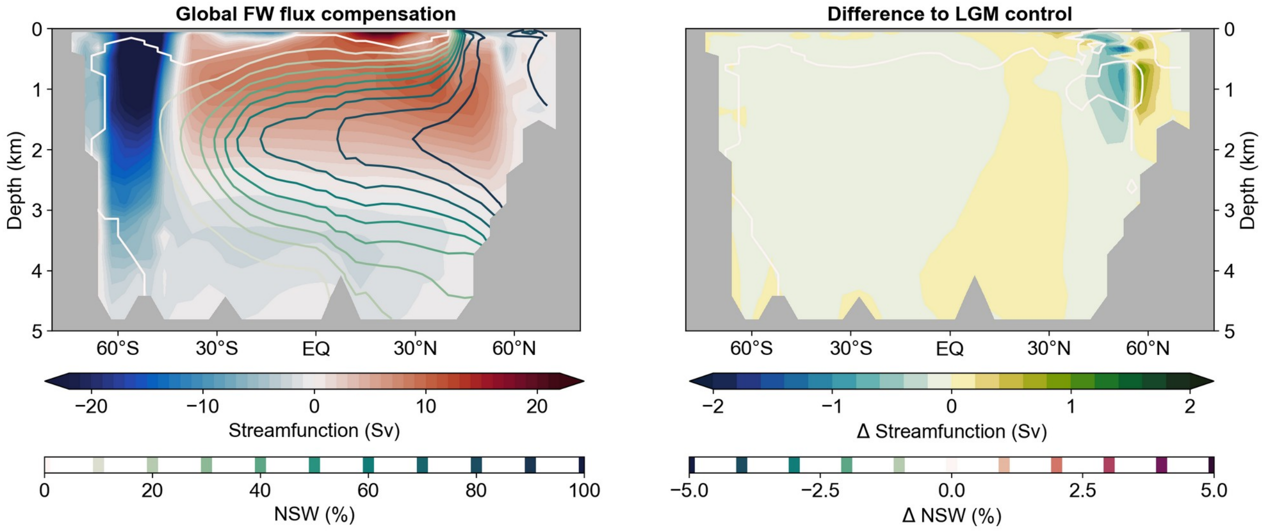

Fig. S9. LGM Sensitivity run for which the freshwater compensation flux was applied uniformly to the global ocean except the Southern Ocean. (*Left*) AMOC stream function and northern-sourced water fraction of the sensitivity run. (*Right*) Difference of left panel to the LGM best-fit simulation as shown in Fig. 2.

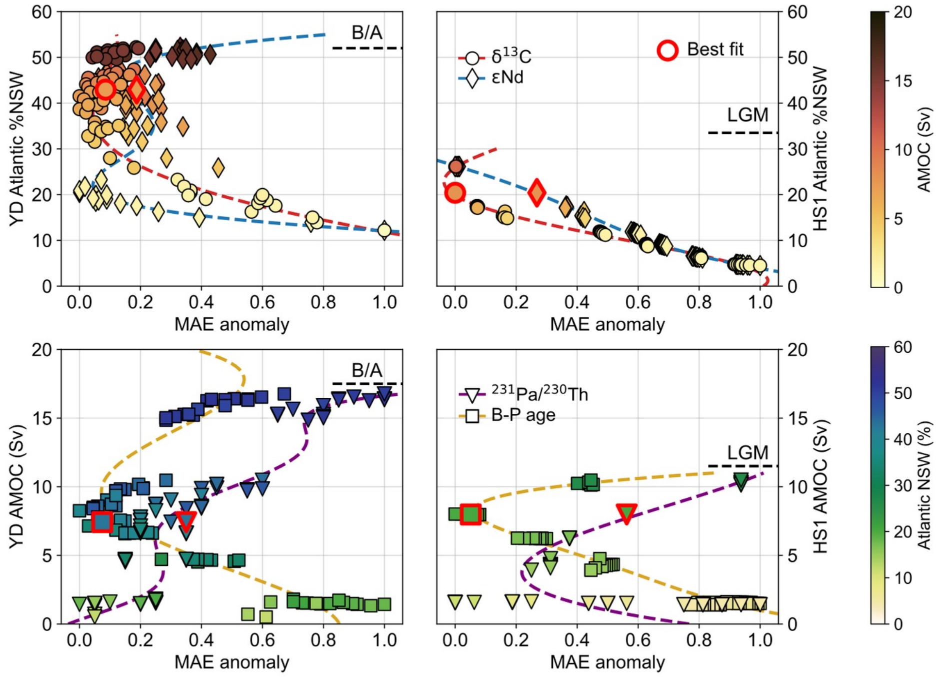

Fig. S10. *(Top)* Normalized cost functions (weighted by LGM residuals (Fig. 1C) and number of records, see Methods for details) of $\delta^{13}C$ and εNd versus the fraction of NSW in the Atlantic during *(left)* the Younger Dryas and *(right)* during Heinrich Stadial 1. *(Bottom)* Same as top but Pa/Th and B-P age versus the minimum AMOC strength during *(left)* the Younger Dryas and *(right)* during Heinrich Stadial 1. Black dashed lines mark NSW fraction and AMOC strength preceding the cold events (i.e., LGM for HS1 and Bølling-Allerød for YD). Colored dashed lines are plotted only as visual aid.

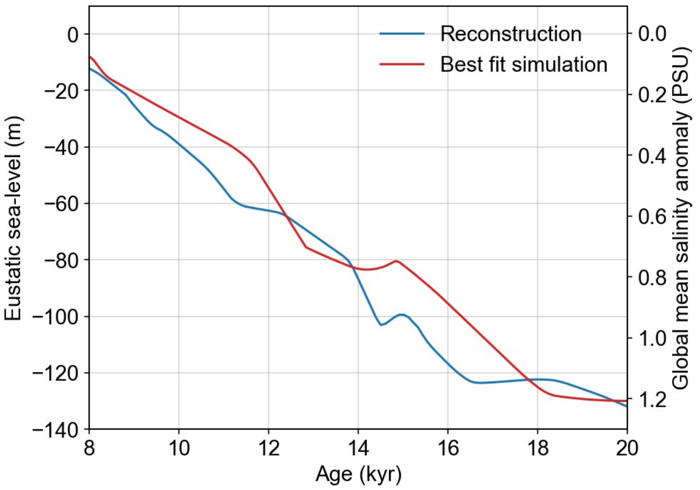

Fig. S11. Eustatic sea-level over the deglaciation as reconstructed by ref.[1] (blue) and the best-fit transient simulation (red). The Bern3D model has a rigid-lid ocean, and global mean salinity anomalies have therefore been here converted to sea-level equivalent.
